# Using DNA barcoding and field surveys to guide wildlife management at Nanjing Lukou International Airport, China

**DOI:** 10.1002/ece3.10005

**Published:** 2023-04-13

**Authors:** Wan Chen, Keer Miao, Yizheng Liu, Jie Zhang, Yang Zhao, Dongfang Hu, Pengcheng Wang, Peng Li, Qing Chang, Chaochao Hu

**Affiliations:** ^1^ College of Environment and Ecology Jiangsu Open University (The City Vocational College of Jiangsu) Nanjing Jiangsu China; ^2^ Jiangsu Key Laboratory for Biodiversity and Biotechnology, School of Life Sciences Nanjing Normal University Nanjing Jiangsu China; ^3^ China Academy of Civil Aviation Science and Technology Beijing China; ^4^ Nanjing Lukou International Airport Nanjing Jiangsu China; ^5^ Analytical and Testing Center Nanjing Normal University Nanjing Jiangsu China

**Keywords:** airport, bird diversity, bird strike, DNA barcode

## Abstract

The conflicts between wildlife and aircraft have increased due to the development of the aviation industry. While many studies have quantified the relative hazards of wildlife to aircraft, few studies have combined DNA barcoding techniques with field surveys of bird communities in different habitats to reveal the exact species involved in bird strikes and how the habitat heterogeneity around airports affects bird communities and even the occurrence of bird strikes. Taking Nanjing Lukou International Airport in China as an example, based on the DNA barcoding technology and detailed field research, we establish the most commonly struck species, which can help managers identify the level of hazard and lead to meaningful reductions in hazards and costs associated with bird strike. The investigation of bird communities showed that there were 149 bird species recorded within an 8 km radius. There were 89, 88, 61, and 88 species in the woodland, wetland, farmland, and urban area, respectively. In total, 303 samples identified 82 species representing 13 orders and 32 family of birds from bird strike cases, of which 24 species were not found in the field survey. Passeriformes were the most common order of birds identified, with 43 species represented in 167 identifications. Skylark, Thrush, Shrike, Lapwing, and Swallow were most likely to cause damage or substantial damage to aircraft when strikes occurred. In addition to birds, we identified 69 bats individuals (accounting for 22.77%) using DNA barcoding. The Bray–Curtis similarity analysis revealed that species involved in bird strike had the highest similarity with urban area. Our findings suggest that policymakers should pay more attention to managing the wetlands and urban areas surrounding the airport. These findings imply that DNA barcoding can add to the environmental monitoring in airports, which can facilitate hazard management and improve air safety.

## Graphical abstract

Using DNA barcoding technology, we identified 82 bird species belonging to 13 orders and 32 families involved in bird strike incidents at Nanjing Lukou International Airport in China. Additionally, we conducted field research and recorded a total of 149 bird species.Interestingly, 24 bird species were observed exclusively in bird strike cases and not during field surveys.

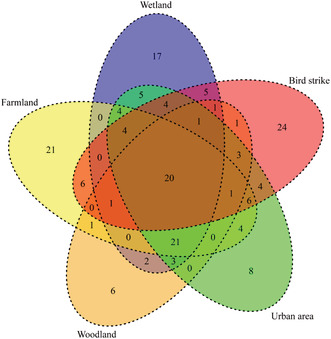

## 1 INTRODUCTION

With the development of the aviation industry, conflicts between wildlife and aircraft have become apparent and reported increasingly, causing huge losses worldwide every year (Jeffery & Buschke, 2019). Bird strikes are frequent worldwide, causing substantial human safety concerns and cause extensive economic losses to the civil aviation industry, which was one of the important threats to aviation safety (Dolbeer et al., 2022; Hu et al., 2020). In order to reduce bird strike accidents, many studies mainly focus on the birds ecology at airports (Blackwell et al., 2019; Fernandez‐Juricic et al., 2018), structure of bird communities (Yuan et al., 2023), bird movements (Nilsson et al., 2021), surrounding environmental governance (Conkling et al., 2018; Zhao et al., 2019), and bird strike risk assessment (Hu et al., 2020). Measures to mitigate this hazard require accurate data about the species involved in these incidents, and the field bird surveys associated with the identification of bird collisions with aircraft have important implications and can help managers identify the level of hazard and prioritize mitigation efforts (Waugh et al., 2011). Knowing the exact species involved in bird strikes is fundamental to any management plan that aims to adequately control this hazard (Dove et al., 2008). However, the dynamics of land use near airports that provide different habitats for birds, how bird communities near airports contribute to bird strike risk, and how to reduce bird strike risk are only beginning to be explored.

Many studies focus on the major factors underlying biodiversity near the airport (Conkling et al., 2018; Soldatini et al., 2010). Biotic or abiotic factors, for instance, crop types, vegetation composition, food availability, and landscape structure have been shown to modify the community composition of wildlife in airports (Alquezar et al., 2020). Still, relatively little attention has been directed to mitigating bird attractants originating from the surrounding landscape so as to reduce strike risk (Coccon et al., 2015). Surveys of bird abundances in associated habitats and airspace over time might compose the risk estimate, or serve in concert with the risk estimate to prioritize wildlife management efforts (Blackwell et al., 2013; Coccon et al., 2015).

To our knowledge, methods to link off‐airport (i.e. within an 8 km radius around an airport), habitat‐specific bird survey data to strike risk associated with an airport or airbase are few. Our purpose was to understand how bird communities using the landscape, might contribute to strike risk and other aircraft operating from the airbase. Identification of bird strikes can be used to assist with accident investigations and support management plans to reduce risks in critical areas, such as airports (Dove et al., 2009). Removing some species and/or the resources used by them from airport sites (e.g. food, water) and the strict control of land use and economic activities in nearby areas may reduce the frequency of bird strikes (Scott et al., 2014).

When biological remains from bird strikes are not suitable for morphological identification, genetic methods can be used to associate unknown samples to a reference sample by comparing DNA sequences that differ between species (Dawnay et al., 2007). DNA barcoding was designed to be a universal system for cataloging and identifying animal species based on standard sequences of the cytochrome oxidase I (COI) gene. The system comprises a reference database with authenticated sequences and a searching tool used for species identification (BOLD; Kerr et al., 2007; Ratnasingham & Hebert, 2007). DNA barcoding has already been used successfully to identify evidence from bird strikes (Gaikwad et al., 2016). Investigating bird use of landscape fields, DNA barcoding from bird strike events and their potential hazards could inform airport personnel of the associated wildlife strike risk adjacent to air operations areas and encourage the establishment of alternative land uses (Conkling et al., 2018; DeVault et al., 2016; Pfeiffer et al., 2018).

In this study, based on a 2‐year continuous survey and bird strike the identification, we explored multiple dimensions of diversity for bird communities in different types of land cover (i.e. with spatial variation across farmland, woodland, wetland, and urban area) in Nanjing Lukou International Airport and compare to the bird strike identifications. Specifically, we first characterized bird community patterns for land cover. Different land covers can lead to the difference in community similarities, depending on available resources. And then these communities were then compared to the bird strike residue data. Our objectives were to (1) quantify the bird community pattern at Nanjing Lukou International Airport; (2) compare bird field survey findings with identification of species involved in bird strikes on aircraft; (3) define the most hazardous groups of species to direct the management efforts.

## 2 MATERIALS AND METHODS

### 2.1 Study area

**FIGURE 1:**
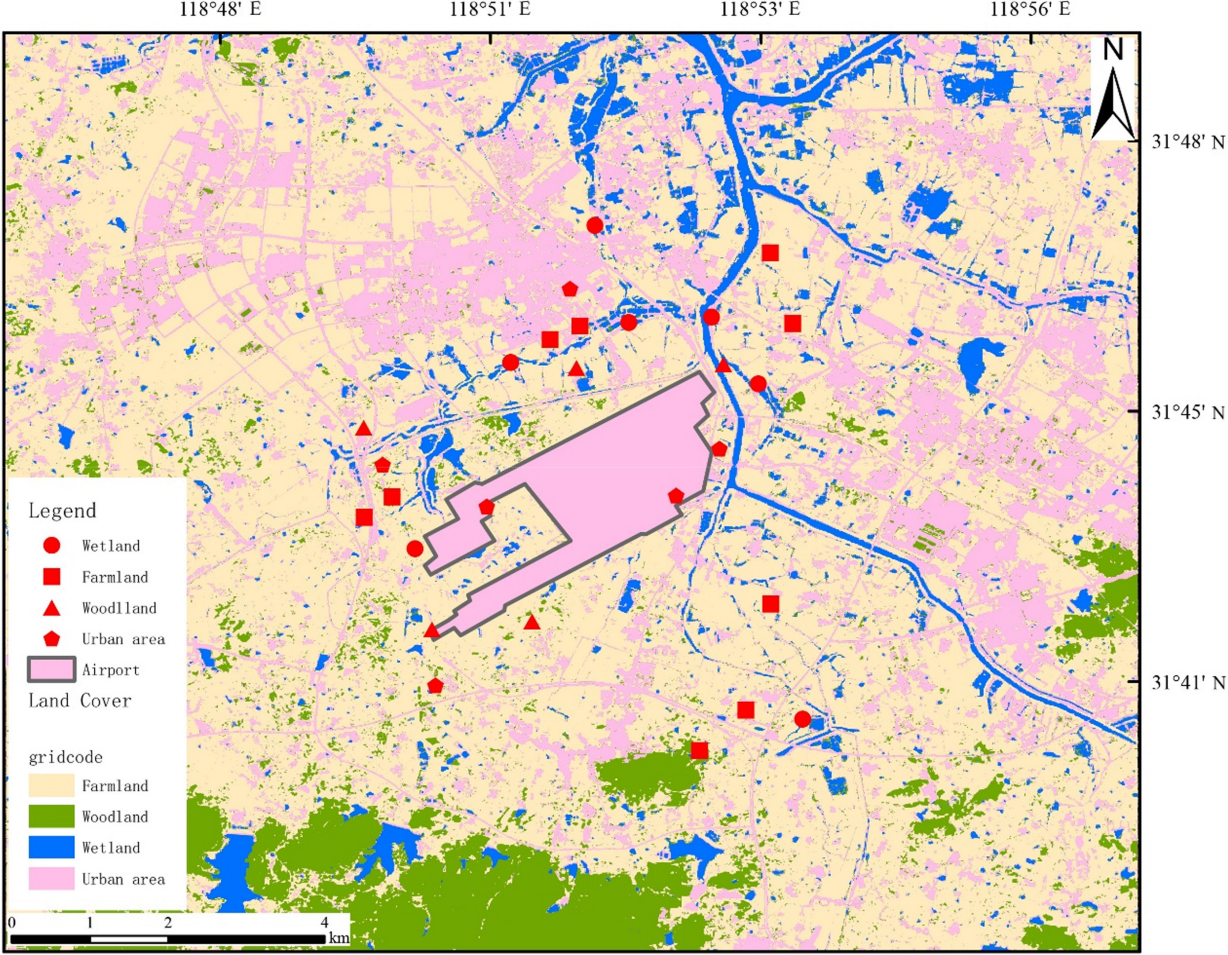
Habitat distribution around Nanjing Lukou International Airport.

Our approach was developed at Nanjing Lukou International Airport (NLIA), located in Nanjing, Jiangsu Province, China (Figure 1). The region experiences a subtropical monsoon climate. Temperatures averaged 15.4°C and precipitation 1106.5 mm below the long‐term averages of 14.98°C and 780 mm during our study, respectively. According to the guidance provided by the International Civil Aviation Organization (ICAO) in the Airport Planning Manual, we considered land uses present within 8‐km of the airfield as hazardous to aviation.

Data collection was carried out from November 2017 to December 2019. Land cover data were obtained to evaluate the effects of landscape matrix types, which were downloaded from the Yangtze River Delta Science Data Center, National Earth System Science Data Center, National Science & Technology Infrastructure of China (http://nnu.geodata.cn:8008) with a resolution of 9 × 9 m per grid cell. Habitats within this radius were classified into four main habitat categories: (1) Farmland, (2) Urban area, (3) Wetland, and (4) Woodland. Approximately 5.85% of the area was wetland, 12.52% was forest, and 22.14% was urban area, while the majority of habitat was farmland (59.49%). These habitats are the most continuous and widely distributed landscape structures and are often considered in birds and landscape studies (Filloy et al., 2019; Girma et al., 2017; Soifer et al., 2021).

Blackwell et al. (2009) encouraged the inclusion of land‐use data around airports and wildlife use in these habitats into wildlife strike risk assessment (Blackwell et al., 2009). The bird species richness and abundance of four habitat types in the study area were investigated using the point transect count method. A total of 27 point transects representing each habitat were systematically established to estimate the diversity and abundance of birds. We randomly selected nine sampling points in farmland, 7 in wetland, 5 in woodland and 6 in the urban area to ensure that the sampling effort on each matrix type was roughly proportional to its area. According to the climatic characteristics of Jiangsu Province, China, the data from March to May are counted as spring surveys, the data from June to August are counted as summer surveys, the data from November to November 2019 are counted as autumn surveys, and the data from December to February are counted as winter surveys.

### 2.2 Bird survey

We documented abundance and landscape use by bird community from November 2017 to December 2019. We used a stratified sampling design to assess the bird community across the four dominant habitat types found in the study area: farmlands, woodlands, wetlands, and urban areas. A point transect count was employed to investigate bird species richness and abundance per habitat type. Bird communities were surveyed monthly within each site using point counts in 20 min. Bird identifications and counting of individuals were conducted by direct observations aided with binoculars (8 × 42). Observations were made by standing in the middle of the point transect and observing 360° round quietly and gently up to a distance of 30 m radius. We recorded all birds seen or heard and excluded fly‐overs from obtaining the data of the entire community, taxonomic information using field guides for bird identification (Zheng, 2017).

To avoid visibility problems, survey of the birds was carried out mainly between 30 min after dawn and 10:00 a.m., and between 3:30 p.m. and 30 min before sunset on fair weather days (i.e. no rainfall, fog, or strong wind). At each observation, bird species were identified, and numbers of individuals observed within the 30‐m radius were recorded on a data sheet prepared for this purpose, along with the type of habitat they are located. In addition, ancillary data, such as elevation above sea level, latitude, and longitude, average vegetation height, and percent slope inclination, were recorded with a GPS and clinometers per plot.

### 2.3 Birdstrike data collection

Samples for DNA barcoding were carried out from November 2017 to December 2021. Bird strike is defined as a collision between a bird and an aircraft in flight, or on a take‐off or landing roll. Data on bird strike events and aircraft movements were provided by the airport management authority, bird species registered in airport survey and bird strike databases were grouped (Zheng, 2017).

We received 303 bird strike samples for identification, which were consisted of dried, or ethanol‐preserved fragments of feathers, muscle, or blood. We also recorded the date of strike, the condition of the sample upon receipt, the date submitted to laboratory, and the date of completed identification. These allowed us to evaluate the amount of time to complete DNA identifications and helped us evaluate the types of bird remains currently received for DNA identification and the associated success rate of DNA identifications.

### 2.4 DNA sequencing

Samples were placed into 1.5 mL tubes for processing using sterile techniques, which were transferred to −20°C in the laboratory for long‐term storage at Nanjing Normal University, Nanjing, China. Total genomic DNA was extracted using commercial DNA isolation kit (QIAGEN) based on the manufacturer's protocol. DNA templates were diluted to 50–100 ng/μL in TE buffer. The genes used for DNA barcoding were the mitochondrial COI and 12S ribosomal RNA (12S rRNA; Waugh et al., 2011; Yang et al., 2010). The polymerase chain reaction (PCR) was carried out in 50 μL volumes containing 5.0 μL 10 × buffer, 1.5 mM MgCl_2_, 0.2 mM each dNTP, 0.2 μM each of primers, 5–10 ng of template DNA and 1.25 U of Taq DNA polymerase (Takara Biotechnology). The PCR cyclings were performed with initial denaturation for 8 min at 95°C, 30 cycles of 40 s at 95°C, 30 s at 52°C, 1 min at 72°C, and a final extension for 10 min at 72°C. The products of PCR were purified using DNA and Gel Band Purification kit (Amersham Biosciences), and then sequenced using the forward and reverse primers on an ABI 377 sequencer. Resulting sequence chromatograms were aligned, checked, edited, and trimmed using the software SeqMan (DNAStar, Inc.).

Consensus sequences were searched in BOLD Species Level Barcode Records database, and NCBI BLAST database. Based on the similarity results and the representativeness of the group in the database, the species can be identified when a query sequence match of >98% was considered a successful identification (Waugh et al., 2011). Taxonomic information and species occurrence data followed A Checklist on the Classification and Distribution of the Birds of China (3rd; Zheng, 2017).

### 2.5 Statistical analysis

We evaluated the bird sampling adequacy and estimate the true species richness with species accumulation curve (Colwell et al., 2004). We calculated species richness, Shannon–Wiener index, Simpson's diversity index, Pielou's evenness index, Bray–Curtis similarity, and the sampling adequacy analysis using the “vegan” package (Oksanen et al., 2019). All data were summarized per plot per habitat types during each season. Considering the non‐normal distribution of the data, we performed the multiple comparison test after the Kruskal–Wallis test to compare the mean species richness and abundance per habitat types per plot among seasons. The significance level was set at *α* = .05. All analyses were performed in R 4.0.1 (R Core Team, 2020).

## 3 RESULTS

### 3.1 Bird survey

From November 2017 to October 2019, a total of 29,168 individuals grouped into 149 bird species were recorded. The species accumulation curve of the bird survey was close to asymptotes, indicating that the data integrity of the bird species survey was high and fully met the requirements of analysis. The five most commonly identified birds with the highest number of individuals were all resident birds. Eurasian tree sparrow (*Passer montanus*) was the most abundant bird species in the study area number 7812 (0.068%). And then, from large to small, they were white‐cheeked Starling (*Spodiopsar cineraceus*), Crested Myna (*Acridotheres cristatellus*), Light‐vented Bulbul (*Pycnonotus sinensis*), Spotted Dove (*Streptopelia chinensis*). The migratory species of Eurasian skylark (*Alauda arvensis*) was the sixth relatively abundant species in the study area (Table S1).

There were 89, 88, 61, and 88 species in the woodland, wetland, farmland, and urban area, respectively (Table 1). Species richness was higher in the woodland matrix than in other matrix types. However, the largest number of species abundance was recorded in urban area and the least in the wetland, which was not consistent with the pattern of species richness.

**TABLE 1:** Bird diversity in the four kind of habitats.

	Farmland	Wetland	Woodland	Urban area
Species richness	61	88	89	88
species abundance	6728	4853	6806	10,781
Shannon–Wiener index	1.18	1.23	1.55	1.44
Simpson's diversity index	0.58	0.6	0.69	0.62
Pielou's evenness index	0.74	0.78	0.77	0.69

### 3.2 Bird strike statistics

From November 2017 to December 2021, total of 303 samples with viable DNA, barcoding identified 82 species representing 13 orders and 32 families of birds from bird strike cases. Passeriformes were the most common order of birds identified, with 43 species represented in 167 identifications, accounting for 72.29% of the total birds. The four most commonly identified taxa associated with birdstrikes were Eurasian Skylark (*Al. arvensis*), Gray‐backed Thrush (*Turdus hortulorum*), Olive‐backed Pipit (*Anthus hodgsoni*), and Brown Shrike (*Lanius cristatus*), which identified in 24, 16, 11, and 10 cases, respectively. Of the species identified, 27.21% were non‐passeriform. Among these bird strikes, one incident caused serious damage to the aircraft, four caused moderate damage, and 14 caused minor damage.

**FIGURE 2:**
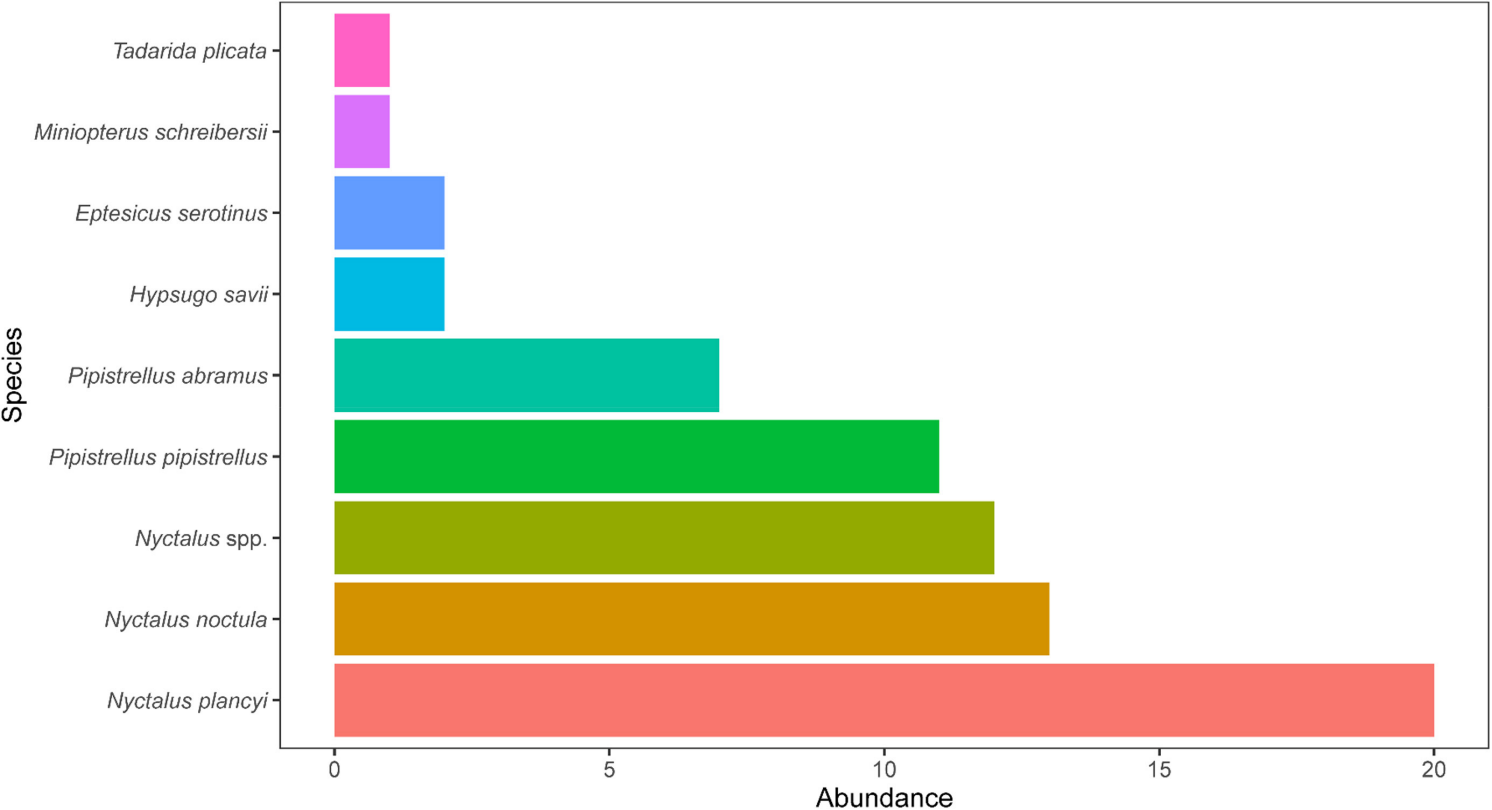
The abundance of bat species based on the bird strike identification.

In addition to birds, we identified 69 bats individuals (accounting for 22.77% of the total) using DNA barcoding, which belongs to eight species, including 20 Chinese Noctule (*Nyctalus plancyi*), 13 Noctule (*Ny. noctule*), 11 Common Pipistrelle (*Pipistrellus pipistrellus*) and 7 Japanese Pipistrelle (*Pi. abramus*; Figure 2). Among these bats, the largest number of bats from the genus *Nyctalus*, occupying 65.22% of the overall bats (Figure 2). These bats data were not included in the following analysis.

### 3.3 Similarity analysis

Bray–Curtis similarity analysis showed that bird communities in urban area and farmland had the highest similarity, with a value of 0.35, followed by farmland and wetland (0.33), and the Bray–Curtis similarity between the rest of the habitats were all lower than 0.30. Regarding the number of common species, woodland has the highest value with urban area (66 species), and then followed by urban area and wetland, the other pairs are all lower than 60 species. The similarity analysis with bird strike residues identified that bird strike events had the highest similarity with urban area (Table 2).

**TABLE 2:** Bray–Curtis similarity index (upper right) and the number of common species (lower left).

	Farmland	Wetland	Woodland	Urban area	Bird strike
Farmland		0.33	0.29	0.35	0.1
Wetland	49		0.21	0.3	0.12
Woodland	44	50		0.3	0.13
Urban area	49	62	66		0.17
Bird strike	28	36	38	43	

**FIGURE 3:**
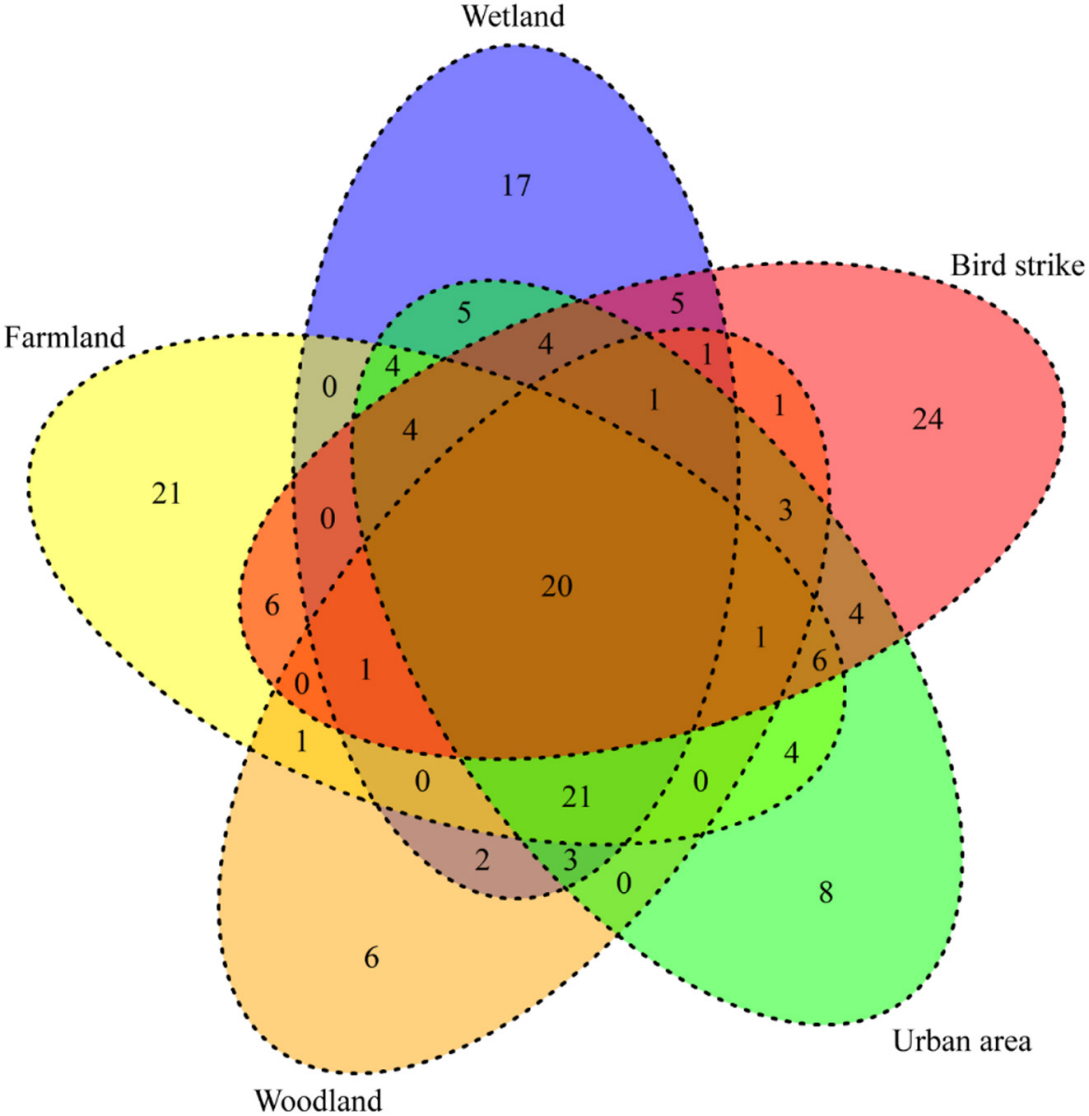
Species overlap of birds among land covers and bird strike incidents, illustrated by a Venn diagram representing the number of species recorded. The colors represent different habitats, with overlapping colors representing common habitats. It is called intersection when multiple circles overlap each other, and represents the number of species in each habitat at the same time.

In total, 24 bird species were not found in the field survey (Figure 3). All of these were migratory birds in the study area. Among them, Siberian Rubythroat *Calliope calliope* occurred bird strikes eight times, Marsh Grassbird *Locustella pryeri* occurred four times, Lanceolated Warbler *Locustella lanceolata* occurred three times, and Great Reed Warbler *Acrocephalus arundinaceus*, White's Thrush *Zoothera aurea*, Siberian Blue Robin *Larvivora cyane* all occurred two times, all the other birds only occurred one bird strike.

### 3.4 Seasonal variation

**FIGURE 4:**
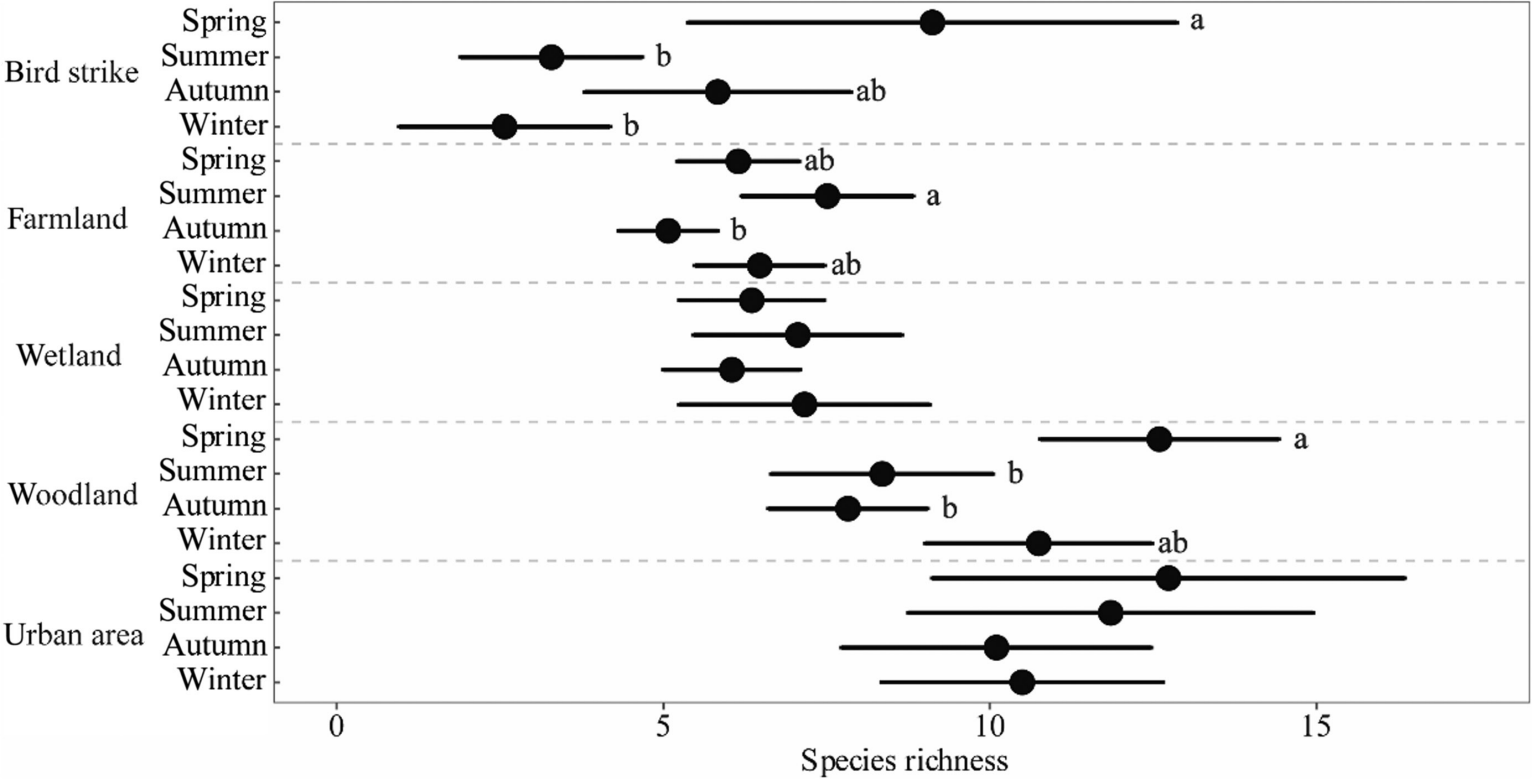
Species richness under each habitat and bird strike events, and comparison of differences with seasons using the Kruskal–Wallis test. The dots represent the average and the short lines represent the 95% confidence interval. Letters after the data represent the results of multiple comparisons, which were adjusted using the Bonferroni correction.

**FIGURE 5:**
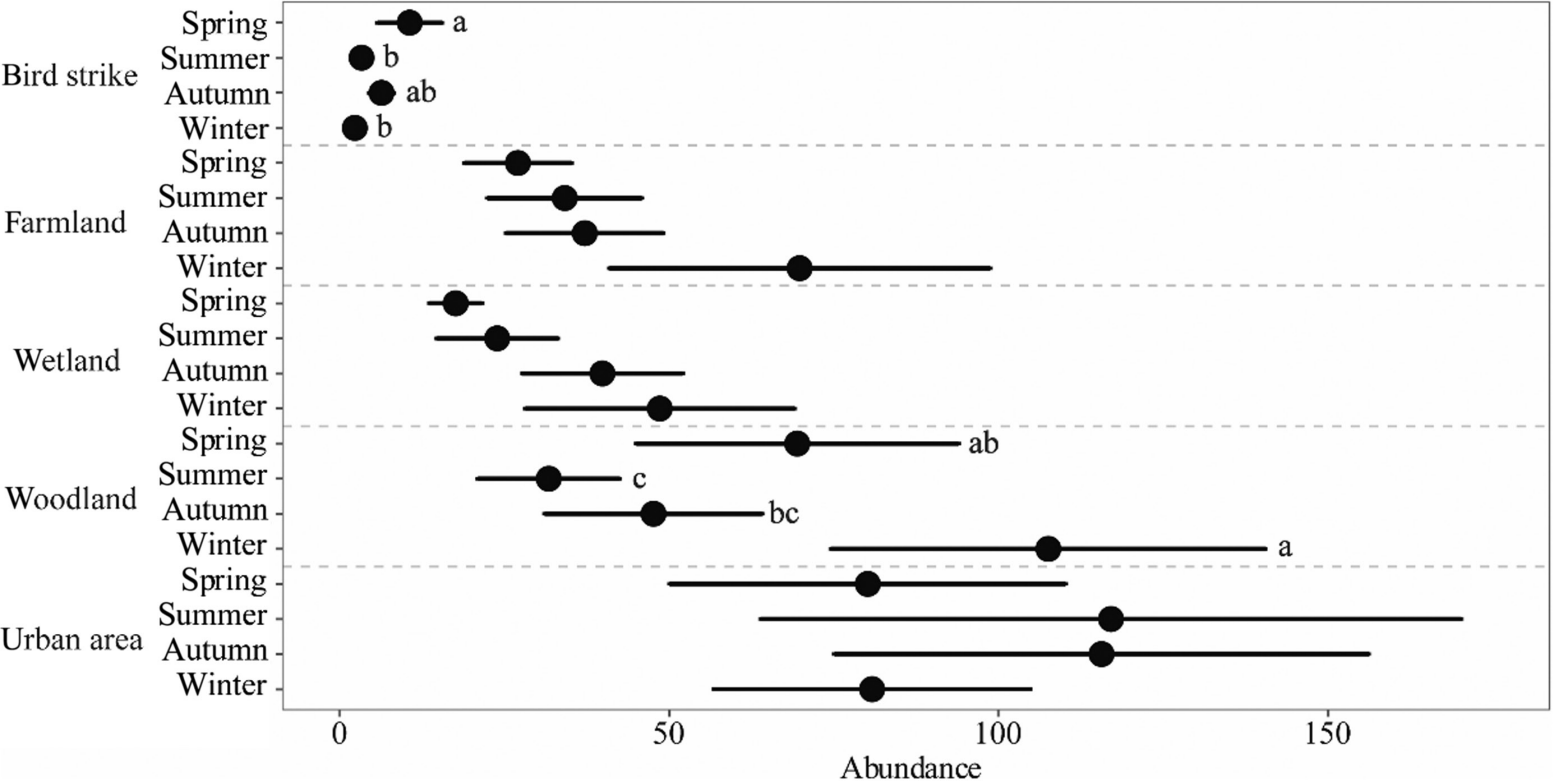
Species abundance under each habitat and bird strike events, and comparison of differences with seasons using the Kruskal–Wallis test. The dots represent the average and the short lines represent the 95% confidence interval. Letters after the data in the figure represent the results of multiple comparisons, which were adjusted using the Bonferroni correction.

Species richness (Kruskal–Wallis test: *χ* ^2^ = 8.432, *p* = .038) and abundance (*χ* ^2^ = 8.432, *p* = .038) differed among the seasons. Species richness were significantly higher in spring than in other seasons. There was a significant difference in the mean species richness among seasons in bird strike event (*χ* ^2^ = 12.353, *p* = .006), farmland (*χ* ^2^ = 7.996, *p* = .046) and woodland (*χ* ^2^ = 18.586, *p* < .001) habitats (Figure 4). As for the relationship between abundance and seasons, there was a significant difference in bird strike event (*χ* ^2^ = 14.569, *p* = .002) and woodland (*χ* ^2^ = 21.819, *p* < .001) habitats (Figure 5).

## 4 DISCUSSION

Nanjing Lukou International Airport is located on the eastern migratory route of migratory birds in China and has a high number of migratory birds and resident birds. The results of this study showed that the highest number of species was from the order of Passeriformes and Charadriiformes, and the highest number of individuals of species were resident birds such as Eurasian Tree Sparrow, White‐cheeked Starling, and Crested Myna. Surveys on airports must be designed using a standardized protocol in order to yield accurate estimates of species abundances, since they are critical to bird strike risk. In this study, we found that habitat type has a significant effect on bird community diversity, bird community in urban area are more of a threat to the wildlife‐aircraft collisions, because bird species differ in different habitats, and species with high bird strike frequency tend to be distributed in different habitats.

The area around Nanjing Lukou International Airport is mainly dominated by farmland ecosystems, and we found 61 species of birds in farmland communities, with a Shannon–Weiner diversity index of 1.18 and Pielou's evenness index of 0.74, which had the lowest species richness. Although farmland is subject to a high level of anthropogenic disturbance, it provides rich and diverse food resources, increasing the species richness and multiplicity of bird communities, and the uniformity is at a high level (Wuczyński, 2016). The present study found 88 species of birds in urban communities, with a Shannon–Weiner diversity index of 1.44 and Pielou's evenness index of 0.69. Urban habitats have some internal heterogeneity and are suitable for a wide range of bird species, but because urbanized environments are mostly similar, habitats tend to filter and select species based on similar characteristics, thus making urbanization tend to homogenize birds and leading to a decrease in community homogeneity (Pellissier et al., 2012). Some bird species are unable to adapt to the urban environment and gradually disappear during urbanization; while the abundance of birds that are able to survive in cities is higher (Sol et al., 2014). Eurasian Tree Sparrow, pigeons, White‐cheeked Starling, and White Wagtail are common birds in urban habitats.

Habitats are relatively less studied in the airport area (Alquezar et al., 2020). To evaluate habitat types influences on community structure, only a few studies indicate that strike rate is positively influenced by the amount of area, close proximity of wetland, and landscape diversity at different extents from airports (Pfeiffer et al., 2018, 2020). The effects of many other landscape attributes on biodiversity surrounding the airport remain poorly known, although they have been assessed in other types (Barros et al., 2019). Bird movement patterns depend on the fractal dimension of agricultural and desert landscapes (Roshier et al., 2008). In urban area, bird diversity is affected by fragmentation and aggregation of the buffer area (Zhang et al., 2021). There are numerous natural and human‐altered environments that serve as habitats and food sources for several high‐risk species posing a risk to aircraft surrounding landscape of the OR Tambo International Airport (Robinson et al., 2021). Hence, a comprehensive understanding of landscape effects on biodiversity is essential to preventing aircraft collisions (Pfeiffer et al., 2020).

Wildlife‐aircraft collisions are notably caused by birds, and the bird community is directly linked to the probability of bird strikes (Blackwell & Wright, 2006). For bird strikes control, many studies pay attention to the species, and they revealed that waterfowls such as ducks and geese cause more damage to aircraft than songbirds, with their density, body mass, and group size significantly influencing the likelihood of aircraft damage (Andersson et al., 2017). Raptors also account for a high proportion of bird strikes (Blackwell & Wright, 2006). However, this study shows that bats are also important risk animals, and they contribute to about 20% of bird strikes in Nanjing Lukou International Airport. Eurasian Skylark (*Al. arvensis* 24/303), Gray‐backed Thrush (*Tu. hortulorum* 16/303), Brown Shrike (*L. cristatu* 10/303), Olive‐backed Pipit (*An. hodgsoni* 10/303), Gray‐headed Lapwing (*Vanellus cinereus* 8/303), Yellow‐browed Warbler (*Phylloscopus inornatus* 8/303), Orange‐flanked Bluetail (*Tarsiger cyanurus* 8/303), Siberian Rubythroat (*C. calliope* 8/303) and Barn Swallow (*Hirundo rustica* 7/303) were struck most often by aircraft beyond airport boundaries. In the United States, waterbirds (cormorants, ducks, geese, and to a lesser extent, gulls) and raptors (including vultures) were most likely to cause damage or substantial damage (DeVault et al., 2016). However, we found that the Skylark, Thrush, Shrike, Lapwing, and Swallow were the most likely to cause damage or substantial damage to aircraft when strikes occurred in Nanjing Lukou International Airport in China.

Surprisingly, we identified 69 bats individuals using DNA barcoding, which accounting for 22.77% of the total bird strike cases, including 20 Chinese Noctule (*Ny. plancyi*), 13 Noctule (*Ny. noctule*), 11 Common Pipistrelle (*Pi. pipistrellus*) and 7 Japanese Pipistrelle (*Pi. abramus*). However, in the United States, birds accounted for 94.8% of attacks and bats just 2.9% (Dolbeer et al., 2022; Dove et al., 2008). Wetlands form critical habitats for bats in urban environments, and urban bat species richness and activity are affected by wetland size, quality, and pollution levels (Mas et al., 2021; Straka et al., 2016). There are many rivers, ponds, and bodies of water around the airport, approximately 5.85% of the area was wetland (Figure 1). The Bray–Curtis similarity analysis revealed that species involved in bird strike had the highest similarity with urban area (Table 2). In urban landscape, bird diversity is affected by fragmentation and aggregation of the buffer area (Zhang et al., 2021). Hence, a comprehensive understanding of landscape effects on biodiversity is essential to preventing aircraft collisions (Pfeiffer et al., 2020). Our findings suggested that policymakers should pay more attention on management of the wetland and urban area surrounding the airport.

## 5 CONCLUSIONS

In this study, we updated information on birds' abundance, habitat use, and bird strake data in the Nanjing Lukou International Airport in China. The field bird surveys associated with identifying bird collisions with aircraft have important implications. This information can help inform practitioners about conservation priorities in areas with high traffic levels by targeting highly vulnerable species, ultimately optimizing limited resources instead of trying to manage species at random. From November 2017 to December 2019, the investigation of bird communities showed that there were 149 bird species recorded. There are 89, 88, 61, and 88 species in the woodland, wetland, farmland, and urban area, respectively. From November 2017 to December 2021, 303 samples identified 82 species representing 13 orders and 32 families of birds from bird strike cases. There were 24 bird species found in the bird strike cases, but not in the field surveys. Passeriformes were the most common order of birds identified, with 43 species represented in 167 identifications. Eurasian Skylark (*Al. arvensis*), Gray‐backed Thrush (*Tu. hortulorum*), Olive‐backed Pipit (*An. hodgsoni*), and Brown Shrike (*L. cristatus*) were the most common passerines identified in 24, 16, 11, and 10 cases, respectively. Surprisingly, we identified 69 bats individuals using DNA barcoding, which accounted for 22.77% of the total bird strike cases, including 20 Chinese Noctule (*Ny. plancyi*), 13 Noctule (*Ny. noctule*), 11 Common Pipistrelle (*Pi. pipistrellus*) and 7 Japanese Pipistrelle (*Pi. abramus*). Skylark, Thrush, Shrike, Lapwing, and Swallow were most likely to cause damage or substantial damage to aircraft when strikes occurred. The Bray–Curtis similarity analysis revealed that species involved in bird strikes had the highest similarity with urban area. Our findings suggested that the policymakers should pay more attention on management of the wetland and urban area surrounding the airport. These findings mean that DNA barcoding combined with bird field surveys could further facilitate risk management and improve aviation safety in environmental monitoring at airports.

## AUTHOR CONTRIBUTIONS

**Wan Chen:** Conceptualization (equal); writing – original draft (equal). **Keer Miao:** Data curation (equal); formal analysis (equal). **Yizheng Liu:** Data curation (equal); formal analysis (equal). **Jie Zhang:** Validation (equal); writing – review and editing (equal). **Yang Zhao:** Funding acquisition (equal); investigation (equal). **Dongfang Hu:** Investigation (equal); methodology (equal). **Pengcheng Wang:** Software (equal); writing – original draft (equal). **Peng Li:** Software (equal). **Qing Chang:** Conceptualization (equal); funding acquisition (equal). **Chaochao Hu:** Conceptualization (equal); data curation (equal); funding acquisition (equal); investigation (equal); writing – original draft (equal); writing – review and editing (equal).

## FUNDING INFORMATION

This research was funded by the DNA sequencing project commissioned by the China Academy of Civil Aviation Science and Technology Research Institute, grant number S13030BY1816; the Natural Science Research of Jiangsu Higher Education Institutions of China, grant number 20KJD180004; Jiangsu Agricultural Biodiversity Cultivation and Utilization Research Center, grant number 100605‐2022‐KY‐00210; and the Jiangsu Academy of Forestry Youth Foundation, grant number JAF‐2022‐01. The funders had no role in study design, data collection and analysis, decision to publish, or preparation of the manuscript.

## CONFLICT OF INTEREST STATEMENT

The authors declare no conflict of interest.

## Supporting information


Table S1

## Data Availability

Data available in article supplementary material (Table S1).
