# Supplementary material for: Using DNA barcoding and field surveys to guide wildlife management at Nanjing Lukou International Airport, China
Source: Ecol Evol. 2023 Apr 13;13(4):e10005. doi: 10.1002/ece3.10005 (PMC10099200; doi:10.1002/ece3.10005)
Supplement: Supplementary file 1 — Table S1 [file ECE3-13-e10005-s001.docx]

Table A1: The bird groups at Nanjing Lukou International Airport.

| English name | Scientific name | IUCN | wetland | woodland | farmland | urban area | bird strike |
| --- | --- | --- | --- | --- | --- | --- | --- |
| Japanese Quail | *Coturnix japonica* | NT |  |  |  | 1 | 1 |
| Chinese Bamboo Partridge | *Bambusicola thoracicus* | LC | 6 | 21 |  | 2 |  |
| Common Pheasant | *Phasianus colchicus* | LC | 5 | 22 | 5 | 20 | 3 |
| Mallard | *Anas platyrhynchos* | LC | 2 | 1 |  | 24 | 3 |
| Indian Spot-billed Duck | *Anas poecilorhyncha* | LC | 2 | 8 | 21 | 70 |  |
| Green-winged Teal | *Anas crecca* | LC | 18 |  |  | 30 |  |
| Little Grebe | *Tachybaptus ruficollis* | LC | 194 | 5 | 12 | 44 | 2 |
| Black-necked Grebe | *Podiceps nigricollis* | LC |  |  |  |  | 1 |
| Rock Pigeon | *Columba livia* | LC | 28 | 48 | 58 | 112 | 2 |
| Oriental Turtle Dove | *Streptopelia orientalis* | LC | 26 | 95 | 109 | 276 | 1 |
| Spotted Dove | *Streptopelia chinensis* | LC | 168 | 392 | 474 | 320 | 1 |
| Grey Nightjar | *Caprimulgus indicus* | LC |  | 1 |  |  |  |
| Fork-tailed Swift | *Apus pacificus* | LC |  |  |  |  | 1 |
| House Swift | *Apus nipalensis* | LC |  |  |  |  | 1 |
| Greater Coucal | *Centropus sinensis* | LC |  |  |  |  | 1 |
| Lesser Coucal | *Centropus bengalensis* | LC |  | 2 |  | 1 |  |
| Common Koel | *Eudynamys scolopaceus* | LC |  | 2 |  | 1 |  |
| Large Hawk Cuckoo | *Hierococcyx sparverioides* | LC |  |  |  | 1 |  |
| Lesser Cuckoo | *Cuculus poliocephalus* | LC |  | 1 |  |  |  |
| Indian Cuckoo | *Cuculus micropterus* | LC |  |  |  | 3 |  |
| Himalayan Cuckoo | *Cuculus saturatus* | LC |  | 1 |  |  |  |
| Common Cuckoo | *Cuculus canorus* | LC |  |  | 1 | 1 | 2 |
| Water Rail | *Rallus aquaticus* | LC | 1 |  |  |  |  |
| Brown Crake | *Zapornia akool* | LC | 8 | 2 | 3 | 6 |  |
| Band-bellied Crake | *Zapornia paykullii* | NT |  |  |  |  | 1 |
| White-breasted Waterhen | *Amaurornis phoenicurus* | LC | 1 |  |  |  |  |
| Common Moorhen | *Gallinula chloropus* | LC | 417 | 15 | 6 | 174 |  |
| Common Coot | *Fulica atra* | LC | 1 |  | 3 |  |  |
| Northern Lapwing | *Vanellus vanellus* | NT | 17 |  |  | 66 |  |
| Grey-headed Lapwing | *Vanellus cinereus* | LC | 28 | 22 | 125 | 213 | 8 |
| Pacific Golden Plover | *Pluvialis fulva* | LC |  |  |  | 5 |  |
| Grey Plover | *Pluvialis squatarola* | LC | 5 |  |  |  |  |
| Long-billed Plover | *Charadrius placidus* | LC | 19 |  |  | 1 | 1 |
| Little Ringed Plover | *Charadrius dubius* | LC | 4 | 1 |  | 30 |  |
| Kentish Plover | *Charadrius alexandrinus* | LC | 2 |  |  | 9 | 2 |
| Greater Sand Plover | *Charadrius leschenaultii* | LC | 2 |  |  |  |  |
| Oriental Plover | *Charadrius veredus* | LC |  |  |  | 4 |  |
| Greater Painted Snipe | *Rostratula benghalensis* | LC | 1 |  |  |  |  |
| Pheasant-tailed Jacana | *Hydrophasianus chirurgus* | LC | 8 |  |  |  |  |
| Eurasian Woodcock | *Scolopax rusticola* | LC |  |  |  | 3 |  |
| Pintail Snipe | *Gallinago stenura* | LC | 1 |  |  | 6 | 4 |
| Swinhoe's Snipe | *Gallinago megala* | LC | 1 |  |  |  | 2 |
| Common Snipe | *Gallinago gallinago* | LC | 1 |  |  | 9 | 1 |
| Whimbrel | *Numenius phaeopus* | LC | 1 |  |  |  |  |
| Spotted Redshank | *Tringa erythropus* | LC | 30 |  |  | 4 |  |
| Common Redshank | *Tringa totanus* | LC | 1 |  |  |  |  |
| Marsh Sandpiper | *Tringa stagnatilis* | LC | 3 |  |  |  |  |
| Common Greenshank | *Tringa nebularia* | LC | 6 |  |  |  | 1 |
| Green Sandpiper | *Tringa ochropus* | LC | 30 | 1 | 11 | 36 |  |
| Wood Sandpiper | *Tringa glareola* | LC | 2 |  | 1 |  | 1 |
| Common Sandpiper | *Actitis hypoleucos* | LC | 6 |  |  |  |  |
| Long-toed Stint | *Calidris subminuta* | LC | 2 |  |  |  |  |
| Yellow-legged Buttonquail | *Turnix tanki* | LC | 1 |  |  | 1 |  |
| Oriental Pratincole | *Glareola maldivarum* | LC |  |  | 5 | 44 | 3 |
| Black-headed Gull | *Chroicocephalus ridibundus* | LC |  |  |  |  | 1 |
| Whiskered Tern | *Chlidonias hybrida* | LC | 1 |  |  |  |  |
| Yellow Bittern | *Ixobrychus sinensis* | LC | 4 | 1 |  | 1 | 2 |
| Von Schrenck's Bittern | *Ixobrychus eurhythmus* | LC |  |  |  |  | 1 |
| Cinnamon Bittern | *Ixobrychus cinnamomeus* | LC | 1 |  |  |  |  |
| Black Bittern | *Ixobrychus flavicollis* | LC | 2 |  |  |  | 1 |
| Black-crowned Night Heron | *Nycticorax nycticorax* | LC | 297 | 119 | 89 | 35 | 2 |
| Striated Heron | *Butorides striata* | LC | 2 |  |  |  | 1 |
| Chinese Pond Heron | *Ardeola bacchus* | LC | 65 | 17 | 83 | 50 | 5 |
| Cattle Egret | *Bubulcus ibis* | NR | 147 | 26 | 337 | 342 |  |
| Grey Heron | *Ardea cinerea* | LC | 4 |  |  |  |  |
| Purple Heron | *Ardea purpurea* | LC |  |  | 1 |  |  |
| Great Egret | *Ardea alba* | LC | 1 |  |  | 5 |  |
| Intermediate Egret | *Ardea intermedia* | LC | 8 | 4 | 1 | 5 |  |
| Little Egret | *Egretta garzetta* | LC | 530 | 72 | 148 | 235 |  |
| Black-winged Kite | *Elanus caeruleus* | LC |  | 1 |  |  |  |
| Oriental Honey Buzzard | *Pernis ptilorhynchus* | LC |  |  |  |  | 1 |
| Chinese Sparrowhawk | *Accipiter soloensis* | LC |  |  | 1 |  |  |
| Japanese Sparrowhawk | *Accipiter gularis* | LC |  |  |  |  | 1 |
| Eurasian Sparrowhawk | *Accipiter nisus* | LC |  | 1 |  |  |  |
| Northern Goshawk | *Accipiter gentilis* | LC |  | 1 |  |  |  |
| Black Kite | *Milvus migrans* | LC |  | 8 |  |  |  |
| Grey-faced Buzzard | *Butastur indicus* | LC |  |  |  |  | 1 |
| Eurasian Buzzard | *Buteo buteo* | LC |  |  |  | 2 |  |
| Collared Scops Owl | *Otus lettia* | LC |  | 3 |  |  |  |
| Oriental Scops Owl | *Otus sunia* | LC |  |  |  | 1 | 6 |
| Little Owl | *Athene noctua* | LC |  | 2 |  |  |  |
| Northern Boobook | *Ninox japonica* | LC |  |  |  |  | 1 |
| Long-eared Owl | *Asio otus* | LC |  | 1 |  |  |  |
| Short-eared Owl | *Asio flammeus* | LC |  | 2 |  |  |  |
| Eastern Grass Owl | *Tyto longimembris* | LC |  |  |  |  | 1 |
| Common Hoopoe | *Upupa epops* | LC | 2 | 7 | 2 | 46 |  |
| Common Kingfisher | *Alcedo atthis* | LC | 1 |  | 2 | 3 |  |
| Pied Kingfisher | *Ceryle rudis* | LC | 8 |  | 3 | 3 |  |
| Grey-capped Woodpecker | *Dendrocopos canicapillus* | LC |  | 1 |  |  |  |
| Great Spotted Woodpecker | *Dendrocopos major* | LC |  | 1 |  |  |  |
| Grey-headed Woodpecker | *Picus canus* | LC |  | 1 |  |  |  |
| Lesser Kestrel | *Falco naumanni* | LC |  |  |  |  | 1 |
| Common Kestrel | *Falco tinnunculus* | LC |  | 2 |  | 12 | 1 |
| Amur Falcon | *Falco amurensis* | LC |  | 2 |  | 71 |  |
| Merlin | *Falco columbarius* | LC |  | 4 |  |  |  |
| Eurasian Hobby | *Falco subbuteo* | LC |  | 3 |  |  |  |
| Peregrine Falcon | *Falco peregrinus* | LC |  |  |  | 1 | 1 |
| Black-winged Cuckoo-shrike | *Lalage melaschistos* | LC |  |  |  |  | 1 |
| Black Drongo | *Dicrurus macrocercus* | LC | 15 | 49 | 17 | 43 | 1 |
| Brown Shrike | *Lanius cristatus* | LC |  | 17 | 16 | 34 | 10 |
| Long-tailed Shrike | *Lanius schach* | LC | 39 | 170 | 98 | 249 | 1 |
| Chinese Gray Shrike | *Lanius sphenocercus* | LC |  | 1 |  |  |  |
| Azure-winged Magpie | *Cyanopica cyanus* | LC | 17 | 86 | 24 | 46 |  |
| Red-billed Blue Magpie | *Urocissa erythroryncha* | LC |  | 1 |  | 1 |  |
| Grey Treepie | *Dendrocitta formosae* | LC |  | 1 |  |  |  |
| Common Magpie | *Pica pica* | LC | 98 | 177 | 136 | 391 |  |
| Great Tit | *Parus major* | LC | 5 | 23 | 1 | 2 |  |
| Chinese Penduline Tit | *Remiz consobrinus* | LC |  |  | 25 |  |  |
| Eurasian Skylark | *Alauda arvensis* | LC | 25 | 160 | 17 | 895 | 24 |
| Oriental Skylark | *Alauda gulgula* | LC | 45 | 13 |  | 217 | 2 |
| Zitting Cisticola | *Cisticola juncidis* | LC | 3 |  |  |  |  |
| Plain Prinia | *Prinia inornata* | LC | 7 |  | 80 | 5 |  |
| Great Reed Warbler | *Acrocephalus arundinaceus* | LC |  |  |  |  | 2 |
| Black-browed Reed Warbler | *Acrocephalus bistrigiceps* | LC | 2 |  |  |  | 2 |
| Lanceolated Warbler | *Locustella lanceolata* | LC |  |  |  |  | 3 |
| Middendorff's Grasshopper Warbler | *Locustella ochotensis* | LC |  |  |  |  | 1 |
| Pallas's Grasshopper Warbler | *Locustella certhiola* | LC |  |  |  |  | 1 |
| Marsh Grassbird | *Locustella pryeri* | NT |  |  |  |  | 4 |
| Barn Swallow | *Hirundo rustica* | LC | 89 | 80 | 220 | 272 | 7 |
| Red-rumped Swallow | *Cecropis daurica* | LC | 36 | 88 | 27 | 147 | 3 |
| Collared Finchbill | *Spizixos semitorques* | LC | 2 | 20 |  | 6 |  |
| Light-vented Bulbul | *Pycnonotus sinensis* | LC | 134 | 878 | 108 | 415 |  |
| Dusky Warbler | *Phylloscopus fuscatus* | LC |  | 2 |  |  | 2 |
| Pallas's Leaf Warbler | *Phylloscopus proregulus* | LC | 3 | 1 |  | 5 | 3 |
| Yellow-browed Warbler | *Phylloscopus inornatus* | LC |  | 4 |  |  | 8 |
| Arctic Warbler | *Phylloscopus borealis* | LC |  | 3 |  |  | 3 |
| Long-tailed Tit | *Aegithalos caudatus* | LC | 66 | 77 | 33 | 3 |  |
| Vinous-throated Parrotbill | *Sinosuthora webbiana* | LC | 75 | 31 | 57 | 7 |  |
| Reed Parrotbill | *Paradoxornis heudei* | NT |  |  | 1 |  |  |
| Masked Laughingthrush | *Garrulax perspicillatus* | LC | 7 | 153 | 4 | 29 |  |
| Crested Myna | *Acridotheres cristatellus* | LC | 517 | 655 | 510 | 790 |  |
| Silky Starling | *Spodiopsar sericeus* | LC | 10 | 103 | 65 | 36 |  |
| White-cheeked Starling | *Spodiopsar cineraceus* | LC | 702 | 897 | 556 | 702 | 1 |
| Black-collared Starling | *Gracupica nigricollis* | LC | 4 | 2 | 23 | 7 |  |
| Common Starling | *Sturnus vulgaris* | LC |  | 3 |  |  |  |
| White's Thrush | *Zoothera aurea* | LC |  |  |  |  | 2 |
| Scaly Thrush | *Zoothera dauma* | LC |  |  |  | 1 |  |
| Grey-backed Thrush | *Turdus hortulorum* | LC |  | 4 |  | 40 | 16 |
| Common Blackbird | *Turdus merula* | LC | 46 | 204 | 104 | 130 |  |
| Eyebrowed Thrush | *Turdus obscurus* | LC |  | 7 |  |  | 1 |
| Pale Thrush | *Turdus pallidus* | LC |  |  |  | 1 | 1 |
| Naumann's Thrush | *Turdus naumanni* | LC |  | 7 |  | 1 | 3 |
| Siberian Blue Robin | *Larvivora cyane* | LC |  |  |  |  | 2 |
| Siberian Rubythroat | *Calliope calliope* | LC |  |  |  |  | 8 |
| Orange-flanked Bluetail | *Tarsiger cyanurus* | LC |  | 1 |  | 2 | 8 |
| Daurian Redstart | *Phoenicurus auroreus* | LC | 14 | 6 | 14 | 7 | 5 |
| Siberian Stonechat | *Saxicola maurus* | NR |  |  | 2 |  | 1 |
| Grey-streaked Flycatcher | *Muscicapa griseisticta* | LC |  |  |  |  | 1 |
| Asian Brown Flycatcher | *Muscicapa dauurica* | LC |  | 1 |  |  | 1 |
| Yellow-rumped Flycatcher | *Ficedula zanthopygia* | LC |  | 1 |  |  |  |
| White-rumped Munia | *Lonchura striata* | LC | 15 | 23 | 23 |  | 1 |
| Eurasian Tree Sparrow | *Passer montanus* | LC | 582 | 1017 | 2566 | 3647 | 3 |
| Western Yellow Wagtail | *Motacilla flava* | LC | 2 |  |  |  |  |
| Gray Wagtail | *Motacilla cinerea* | LC | 5 | 1 | 3 | 1 |  |
| White Wagtail | *Motacilla alba* | LC | 58 | 29 | 84 | 160 | 2 |
| Richard's Pipit | *Anthus richardi* | LC |  |  |  | 12 |  |
| Paddyfield Pipit | *Anthus rufulus* | LC |  |  | 2 |  |  |
| Olive-backed Pipit | *Anthus hodgsoni* | LC | 19 | 53 | 208 | 55 | 10 |
| Brambling | *Fringilla montifringilla* | LC |  | 456 |  | 34 | 3 |
| Chinese Grosbeak | *Eophona migratoria* | LC | 19 | 251 | 26 | 17 |  |
| Japanese Grosbeak | *Eophona personata* | LC |  | 1 |  |  |  |
| Grey-capped Greenfinch | *Chloris sinica* | LC | 4 | 39 | 110 | 9 | 1 |
| Eurasian Siskin | *Spinus spinus* | LC | 2 | 3 |  | 4 |  |
| Tristram's Bunting | *Emberiza tristrami* | LC |  |  | 1 | 1 | 1 |
| Chestnut-eared Bunting | *Emberiza fucata* | LC | 2 |  | 1 |  |  |
| Little Bunting | *Emberiza pusilla* | LC | 12 |  | 1 | 6 | 2 |
| Yellow-browed Bunting | *Emberiza chrysophrys* | LC |  | 3 |  |  | 2 |
| Rustic Bunting | *Emberiza rustica* | VU |  | 7 |  | 1 | 2 |
| Yellow-throated Bunting | *Emberiza elegans* | LC |  | 10 | 7 |  |  |
| Chestnut Bunting | *Emberiza rutila* | LC |  |  |  |  | 1 |
| Black-faced Bunting | *Emberiza spodocephala* | LC | 50 | 68 | 56 | 21 | 5 |
| Pallas's Bunting | *Emberiza pallasi* | LC | 1 |  |  |  |  |
| Reed Bunting | *Emberiza schoeniclus* | LC |  |  | 1 |  |  |
